# Ventilation Performance Evaluation of a Negative-Pressurized Isolation Room for Emergency Departments

**DOI:** 10.3390/healthcare10020193

**Published:** 2022-01-19

**Authors:** Fujen Wang, Indra Permana, Citra Chaerasari, Kwowhei Lee, Tongbou Chang, Dibakar Rakshit

**Affiliations:** 1 Department of Refrigeration, Air Conditioning and Energy Engineering, National Chin-Yi University of Technology, Taichung 411, Taiwan; citra.chaerasari19@gmail.com; 2 Graduate Institute of Precision Manufacturing, National Chin-Yi University of Technology, Taichung 411, Taiwan; indra.refrigeration@gmail.com; 3 Yuanlin Christian Hospital, Changhua 510, Taiwan; 94692@cch.org.tw; 4 Department of Mechanical and Energy, National Chiayi University, Chiayi 452, Taiwan; tbchang@mail.ncyu.edu.tw; 5 Department of Energy Science and Engineering, Indian Institute of Technology Delhi, New Delhi 110016, India; dibakar@iitd.ac.in

**Keywords:** infection control, isolation room, computational fluid dynamics, ventilation performance

## Abstract

Due to the emergence of COVID-19 becoming a significant pandemic worldwide, hospitals are expected to be capable and flexible in responding to the pandemic situation. Moreover, as frontline healthcare staff, emergency department (ED) staff have a high possibility of exposure risk to infectious airborne. The ED isolation room will possibly and effectively isolate the infected patient, therefore safekeeping frontline healthcare staff and controlling the outbreak. However, there is still limited knowledge available regarding isolation room facilities specifically for the emergency department. In this study, field measurement is conducted in an ED isolation room located in Taiwan. CFD simulation is employed to simulate and investigate the airflow and airborne contaminant distribution. Instead of high air-change rates (ACH) that purposes for dilution, this study proposes the arrangement of exhaust air grilles to improve the contaminant removal. The results reveal that the exhaust air grille placed behind the patient’s head is optimized to dilute airborne contaminants.

## 1. Introduction

A new emerging novel virus (SARS-CoV-2) that caused Coronavirus disease 2019 (COVID-19) was first detected in late 2019, with the possible risk to be transmissible through respiratory droplets and direct contact [1]. In March 2020, the World Health Organization (WHO) declared COVID-19 to be a global pandemic [2]. Hospitals are expected to be flexible in responding to these pandemic situations while still providing treatment for other diseases. As frontline healthcare workers, emergency department (ED) staff are responsible for identifying and isolating patients who get symptoms of the COVID-19. Moreover, healthcare workers and other patients within ED have a high possibility of getting exposed to infectious airborne droplets [3]. By placing an isolation facility as close as possible to both the triage space and the ambulance entry, a hospital can minimize its time to isolate a patient after diagnosis, reducing exposure to other patients and healthcare workers [4]. Managing patients in the ED has been proven to be effective at safekeeping frontline healthcare workers from contracting the disease themselves and controlling the outbreak for effective infection control [5]. However, there is still limited knowledge available regarding the isolation room facilities specifically for the emergency department.

The isolation room needs to utilize negative air pressure and dedicated exhaust systems to prevent airborne contaminants from traveling outside the ED. The negative air pressure can be achieved by exhausting more air, which is around 10% larger than the supply air volume [6]. The anteroom usage acts as an airlock to prevent airborne contaminants from the room to the adjacent spaces. Isolation room facility within ED plays a vital role in limiting the outbreaks of infectious disease. Thus, the design must align with the latest code requirements guidelines and best practices. Several guidelines and recommendations for isolation rooms have been published and applied in numerous countries. ANSI/ASHRAE/ASHE Standard-170 provides some guidelines for ventilation systems in healthcare facilities, including isolation rooms [7]. Pressurization, ventilation rate, and filtration are the critical roles to maintain negative-pressurized isolation rooms. CDC Taiwan has recommended that the air-change rate per hour (ACH) be maintained between 8 and 12 ACH, with a differential pressure of at least 8 Pa to the adjacent spaces [8].

Computational fluid dynamics (CFD) simulations act as tools to simulate and predict the airflow as well as the contaminant airborne exhaled by a patient in the isolation room. The solution given by CFD to these predictions is quite effective as it can overcome the limitations of the experimental method of direct measurement [9]. Thus, it will help to improve ventilation performance and contaminant removal. Based on CFD numerical analysis, several studies have been discussed related to airflow pattern and contaminant distribution in a negative-pressurized isolation room. A numerical study on the ventilation strategy to find the effective design in removing the contaminant has been conducted by Cheong and Phua [10]. They found that the exhaust grille should be located near the infectious source at a low level. Furthermore, the location of supply air across the patient’s bed and exhaust grille over the patient’s head in the patient wards creates the effective contaminant control that has been presented by Khankari [11]. Another study on ventilation arrangement has been studied by Kao and Yang [12]. The results of their numerical study show that the parallel directional airflow pattern is the most effective to contain virus droplets. An experimental study on the mock-up isolation room with different negative pressure differential and ventilation rates has been discussed by Tung et al. [13]. The results show that the best ventilation efficiency has the pressure differentials at −15 Pa; using higher ventilation rates and more pressure differential were found to be effective in extracting the contaminant.

The indoor ventilation efficiency could be assessed and evaluated by the tracer gas method in order to achieve a good performance environmental condition. Particle experiments and tracer gas experiments are widely used. However, most researchers simplify the pathogen as particles or gas without considering its biological characteristics [14]. Wang et al. [15,16] used CO_2_ concentration as a pollutant to assess the isolation room performances. The CO_2_ was injected into the isolation room and then monitored until the lower concentration level approached the CO_2_ background concentration level, same as the outdoor environmental condition. In addition, Shih et al. [17] investigated the airflow distribution and concentration decay of a negative-pressurized biosafety laboratory. Through CFD simulation, it was revealed that ventilation performance could be improved just by relocating the supply air and outlet air. Other research revealed that the arrangement could help the control of bacterial concentration in the isolation room.

In this study, field measurement was conducted in an emergency department isolation room located in Taiwan. The results from the field measurement were used as input for boundary conditions in the CFD numerical simulation in order to evaluate the ventilation performance. Therefore, the arrangement of exhaust air grilles was proposed to find a suitable location for contaminant removal.

## 2. Methodology

### 2.1. System Description

**Figure 1:**
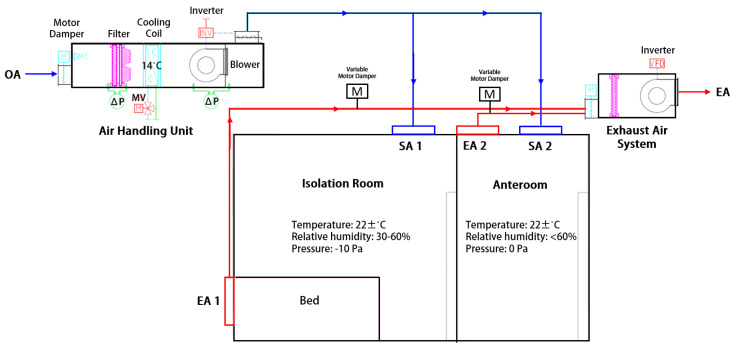
The schematic diagram of the emergency department isolation room.

The total volume of the isolation room is around 16.9 m^3^. The schematic diagram of the ventilation system is shown in Figure 1. The ceiling diffuser is placed near the door entrance of the isolation room and across the patient’s bed to distribute conditioned supply air inside the room. Meanwhile, the exhaust air grille is located at lower levels of the right-side wall at length. The anteroom is provided adjacent to the ED isolation room; nevertheless, this research only evaluated the ventilation system in the isolation room.

The ED isolation room should be designed accordingly based on the standard to provide adequate ventilation at 12 ACH and maintain constant volume to provide consistent ventilation in the room. Fresh air will be conditioned inside an air handling unit (AHU), then it will be supplied to the anteroom and the ED isolation room through ceiling diffusers. The temperature of the supply air should be at 22 ± 2 °C to ensure the thermal comfort of the patient [18]. Lastly, the exhaust air will be removed through the exhaust air grilles. The pressure differential of the isolation room should be kept at −8 Pa with its adjacent anteroom. Pressure control is maintained by modulating the main supply and exhaust dampers based on pressure transducer signals inside the isolation room. The pressurization specification can be achieved by exhausting at least 15% more air than the supply air [19]. Variable frequency driven (VFD) for the exhaust fan is used to adjust the speed.

### 2.2. Field Measurements

The field measurement data were used for input of boundary conditions to conduct the CFD simulations. The result data of airflow rate and pressure test from the measurement are shown in Table 1. ACH can be calculated from the estimated diffuser airflow rate divided by the room’s volume, which results in 16.76 ACH. Therefore, the isolation room has complied with the CDC guidelines of Taiwan [20]. Meanwhile, the ED isolation room was maintained at a pressure of −10.2 Pa to the anteroom.

### 2.3. CFD Simulation and Improvement Strategy

**Figure 2:**
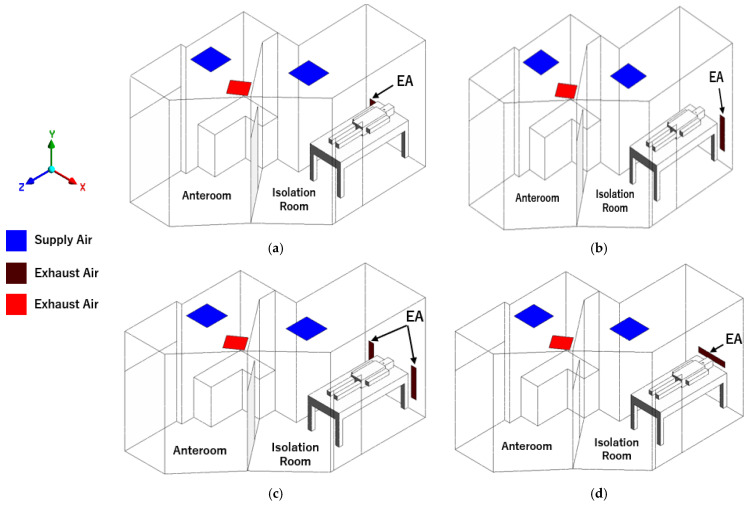
The geometry model of the isolation room based on ventilation strategies. (**a**) Case 1: baseline case. (**b**) Case 2: reverse exhaust air grille. (**c**) Case 3: two exhaust air grilles beside patient’s bed. (**d**) Case 4: exhaust air grille behind patient’s head.

The CFD simulation of the investigated ED isolation room was performed by ANSYS Fluent Workbench Version 2020 R2 [21]. ANSYS fluent provides several equations to solve problems, including laminar and turbulent fluid flow problems, and incompressible and compressible fluid problems. The 3D geometry model of the ED isolation room is created based on the existing layout, as shown in Figure 2. ASHRAE standard and CDC guidelines [7,8] have instructed that exhaust grilles shall be located at the low levels on the wall or the wall near the head of the bed for infection control purposes. The original design may not be the best or most suitable location for the exhaust air grilles. Therefore, the exhaust grilles arrangement strategy was conducted to improve ventilation performance and find suitable contaminant removal locations. Thus, it will reduce the risk of contaminant exposure for the healthcare staff. Four cases were evaluated in this study to improve the ventilation system as described below.

- Case 1: left wall-mounted exhaust air grille located about 300 mm above the floor. It will be the baseline case (Figure 2a).
- Case 2: right wall-mounted exhaust air grille, the reverse of case 1 (Figure 2b).
- Case 3: two wall-mounted exhaust air grilles are located beside the patient’s head. The total exhaust airflow rate is equal to the baseline case (Figure 2c).
- Case 4: wall-mounted exhaust air grilles located behind the patient’s head at 1000 mm above the floor (Figure 2d).

### 2.4. Boundary Condition

The turbulent model used in this study is a realizable k-ε turbulence model where the flow features include strong streamline curvature, vortices, and rotation. In addition, some researchers have proven that this model provides the best performance of all the k-ε models [22]. The modeled transport equations for k and ε in the realizable k-ε model are written in Equations (1)–(3). $G_{k}$ represents the generation of turbulence kinetic energy due to the mean velocity gradients; $G_{b}$ is the generation of turbulence kinetic energy due to buoyancy; $Y_{M}$ is the contribution of the fluctuating dilatation in compressible turbulence to the overall dissipation rate; $C_{2}$ and $C_{1\varepsilon }$ are constants; and $\sigma_{k}$ and $\sigma_{\varepsilon }$ are the turbulent Prandtl numbers for *k* and ε, respectively. $S_{k}$ and $S_{\varepsilon }$ are user-defined source terms.

(1) $$\frac{\partial }{\partial }\left(\rho k\right)+\frac{\partial }{\partial x_{j}}\left(\rho ku_{j}\right)=\frac{\partial }{\partial x_{j}}\left[\left(\mu +\frac{\mu_{t}}{\sigma_{k}}\right)\frac{\partial k}{\partial x_{j}}\right]+G_{k}+G_{b}-\rho \varepsilon -Y_{M}+S_{k}\;,$$

(2) $$\frac{\partial }{\partial t}\left(\rho \varepsilon \right)+\frac{\partial }{\partial x_{j}}\left(\rho \varepsilon u_{j}\right)=\frac{\partial }{\partial x_{j}}\left[\left(\mu +\frac{\mu_{t}}{\sigma_{\varepsilon }}\right)\frac{\partial \varepsilon }{\partial x_{j}}\right]+\rho C_{1}S_{\varepsilon }-\rho C_{2}\frac{\varepsilon^{2}}{k+\sqrt{v\varepsilon }}+C_{1\varepsilon }\frac{\varepsilon }{k}C_{3\varepsilon }G_{b}+S_{\varepsilon }$$

(3) $$C_{1}=max\left[0.43,\frac{\eta }{\eta +5}\right]\;,\;\eta =S\frac{k}{\varepsilon }\;,\;S=\sqrt{2S_{ij}S_{ij}}$$

In this study, transient simulation was conducted to analyze the decreasing contaminant concentration, with a total simulation time of 500 s. A tracer gas concentration decay method was used to evaluate the ventilation performance [23]. The CO_2_ was selected as a tracer gas, and it was injected into the spaces. The initial contaminant concentration in the ED isolation room was set to 1000 ppm, which was the highest CO_2_ concentration inside the room [24]. Then the CO_2_ was monitored until it reached the desired concentration level approaching the CO_2_ concentration background level of 400 ppm [25]. The patient exhaled a CO_2_ concentration that was assumed to be the contamination source inside the isolation room, at around 45,000 ppm [26]. By monitoring the accumulated CO_2_ inside a room, the ventilation system was assessed sufficiently. The elevated concentration increased other indoor contaminant concentrations. The detail of boundary conditions for the ED isolation room are listed in Table 2.

### 2.5. Grid Independence Test

**Figure 3:**
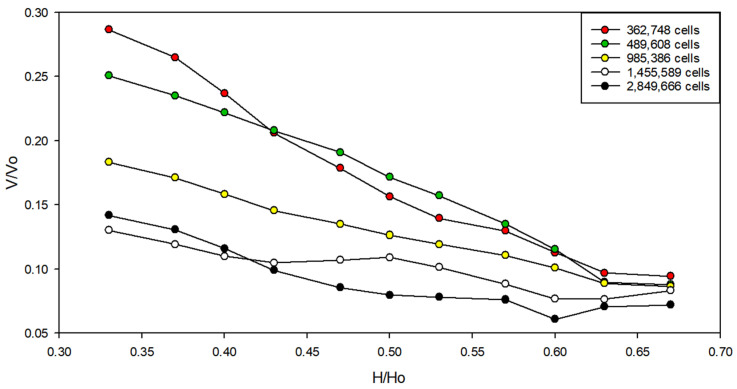
The grid independence test.

The grid independence test was carried out to find the optimum grid size and the number of elements from the meshing process. In this study, the grid-independence study was performed based on each selection of grids number, which is presented in Figure 3. The finite control volume method divides the computational domain into small cells with a tetrahedral grid until grid independence of the dimensionless velocity is achieved. The dimensionless velocity was evaluated at the monitored points in each selection of grids number based on the grid study by Tung et al. [27]. Five grid cells (362,748 cells; 489,608 cells; 985,386 cells; 1,455,589 cells; and 2,849,666 cells) were investigated in this study to perform grid independence test. The velocity magnitude at a specific location close to the patient is referred to as *V*. The maximum velocity of the supply air as a velocity inlet in boundary condition is referred to as *Vo*. The height of the specific location monitored near the patient is referred to as *H*. The height of the isolation room is referred to as *Ho*. The dimensionless velocity of the 362,748 cells and 489,608 cells was quite high compared to the others cells’ number, while the 985,386 cells were quite close to the results of the higher number of cells. The results show no significant change in dimensionless velocity beyond the grid number of 1,455,589 cells and 2,849,666 cells.

Relative error percentage using different grids number results are shown in Table 3. The dimensionless velocity in grids of 1,455,589 cells was quite close to grids of 2,849,66 cells, with relative error at 1.83%. Moreover, increasing the number of grids makes the simulation results more precise for the following range of grids [28]. The grid-independent test proves the numerical robustness of CFD simulation in solving this problem. Therefore, the number of 1,455,589 cells was selected to save the computational time employed for the simulation.

## 3. Results and Discussion

### 3.1. Airflow Distribution

**Figure 4:**
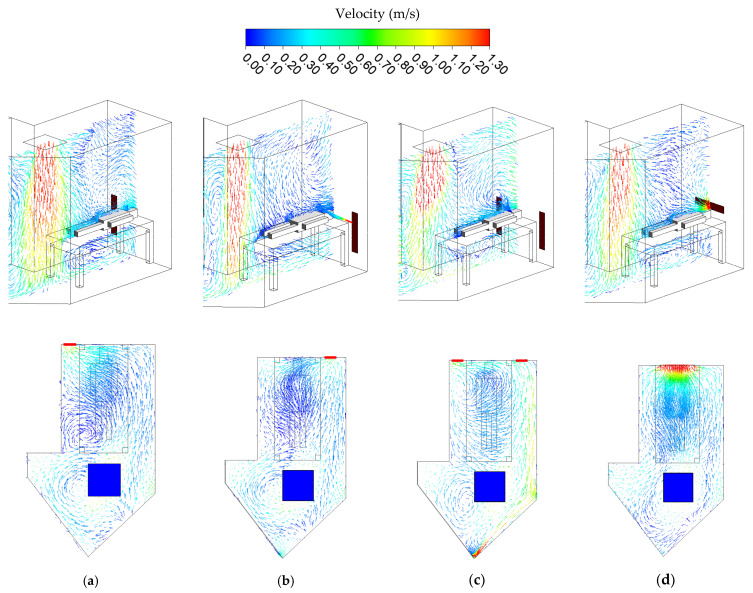
The results of airflow distribution in different cases. (**a**) Case 1. (**b**) Case 2. (**c**) Case 3. (**d**) Case 4.

The result of airflow distribution is presented in the configuration of velocity vectors, simultaneously illustrated in Figure 4 with the isometric plane and top plane at the height of 1.2 m above the floor. The laminar airflow distribution develops downward after setting off the air supply, and it spreads out thoroughly across the isolation room. Indicated in Figure 4a, Case 1 is a baseline case in which most of the airflow is suffocated into the exhaust air located on the left side of the bed because the force of negative pressure is too high. A vortex velocity field is established at the top of the patient’s head, resulting from exhaled air from the patient. Another vortex field is also established near the left side of the ceiling. The vortex velocity field highlights the possibility of assembling contaminants in that zone. Contrarily, in case 2, most of the airflow is suffocated into the exhaust air located on the right side of the bed (Figure 4b). Nevertheless, a more prominent vortex velocity is found above the patient’s head. Close by the left side of the ceiling, the vortex field becomes more stagnant air. Case 3 indicates fewer of the airflows went out to the exhaust grille on the right side and mostly went to the left side (Figure 4c). Vortex velocities are formed in regions near the entrance door and the ceiling. Similarly, case 4 shows a vortex field above the patient’s head because of the exhaled air pathway (Figure 4d). A higher velocity could be found near the patient’s head that could be possibly brought and directly diluted via the airborne contaminant through the exhaust air grille.

### 3.2. Temperature Distribution

**Figure 5:**
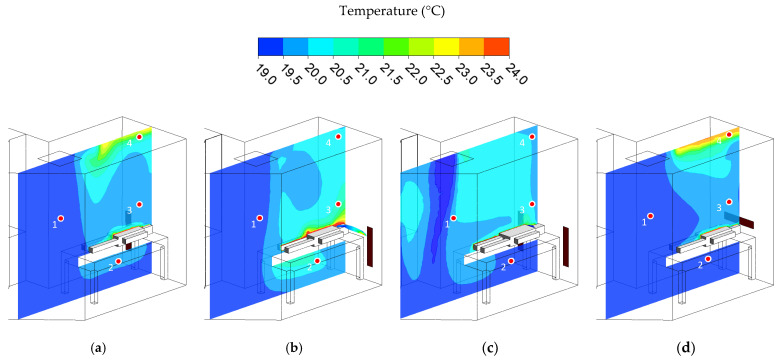
The results of temperature distribution in different cases. (**a**) Case 1. (**b**) Case 2. (**c**) Case 3. (**d**) Case 4.

The temperature distribution is presented in color contour, as illustrated as well in Figure 5. The generating heat comes from the patients, which generated a heat flux at 46.52 W/m^2^k. Meantime, the walls and door are assumed in adiabatic conditions without generating any heat. Consequently, thermal plumes are created around the patient’s body, adding 4 points in the plane located at (1) the middle of the isolation room under the supply air, (2) under the patient’s bed, (3) above the patient’s head and also at the center of the exhaust air, and (4) near the ceiling of the isolation room right on the top of the patient’s head, to perceive the temperature uniformity in the isolation room. Table 4 shows the results of the temperature monitored in four-point locations. Case 1 has an average temperature of 19.82 °C. A moderately higher temperature was only established near the ceiling. Case 2 has an average temperature of 20.07 °C. The temperature distribution is moderately higher near the patient’s head due to the return airflow passing through this zone. Case 3 has an average temperature of 20.14 °C. Case 3 has higher temperature distribution than case 2 and case 1 by 0.3 °C. This may be due to two exhaust air grilles being beside the patient’s bed. As a result, it will remove more conditioned supply air from the ceiling diffuser. Case 4 has an average temperature of 19.89 °C. Overall, case 1 has the lowest compared to the other cases. Still, all of the cases complied with the standard guidelines of healthcare facilities, especially isolation room design.

### 3.3. Concentration Distribution

**Figure 6:**
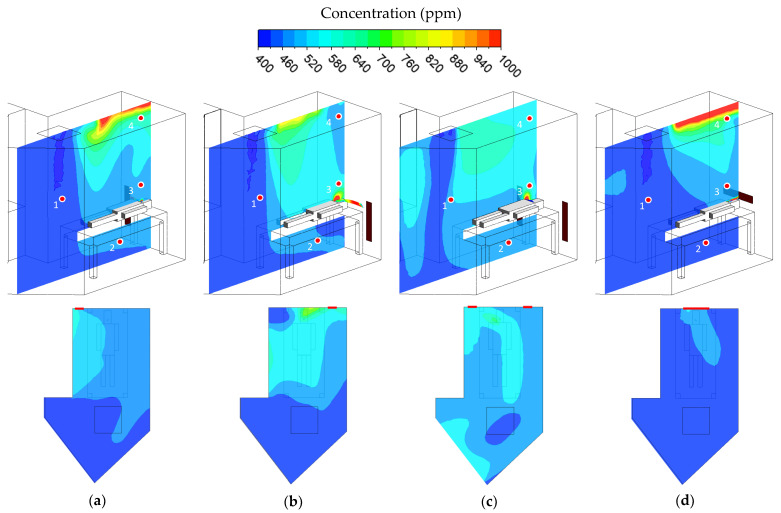
The concentration distribution in different cases. (**a**) Case 1. (**b**) Case 2. (**c**) Case 3. (**d**) Case 4.

The CO_2_ concentration distribution results in different cases are shown in Figure 6. The four monitoring points have analyzed the results of the concentration. Point 1 is located below the supply air at the height of 1.5 m above the floor. This point will result in a lower concentration than other monitoring points because the supply air distributes the fresh air, with a concentration of around 400 ppm, similar to the outdoor CO_2_ concentration background. Point 2 is located behind the patient’s bed at a height of 0.5 m above the floor. Point 3 is located above the patient’s head at a height of 1.2 m above the floor, to monitor the exhalation concentration. Point 4 is located near the ceiling at a height of 2.2 m. Table 5 shows the results of the concentration monitored in four-point locations.

Case 1 as a baseline case presents a satisfactory result. However, the baseline case is not optimal for removing the concentration. Point 1 has a lower concentration than other points due to being located behind the supply air. There are no significant changes with the others. The lowest concentration in point 1 is present in case 1, with 400.15 ppm, while the highest concentration is present in case 3, with 407.33 ppm. In addition, monitoring point 2 is located behind the patient’s bed. A higher concentration behind the patient’s bed occurred in case 1, case 2, and case 3, with concentration values of 468.45 ppm, 471.35 ppm, and 478.59 ppm, respectively. It could also have been caused by the airflow pattern accumulated behind the bed attracted by the exhaust air grille. Other than that, case 4 has a lower and distributed concentration behind the patient’s bed, with a concentration of 432.35 ppm.

Higher CO_2_ concentration accumulated above the patient’s head, indicating the exhaled air pathway, is monitored in point 3. A higher contaminant could be found in the corner left of the ceiling because the vortex field occurs within this region. Case 4 present the lowest concentration in that point. The position of the exhaust air grille in case 4 makes the concentration directly removed. Higher concentrations only occur around the patient’s mouth region, then directly exit through the exhaust air grille behind the head. This indicates that there will not be recirculation of exhaled air from the patient into the supply airstream. Except for than other cases, the released CO_2_ by the patients tends to get spread out in the arrangement of ventilation for case 1, case 2, and case 3. Higher concentration in the ceiling occurs in case 1 and case 4. However, it did not become a critical infected area. The accumulated contaminant in the ceiling will reduce and result in a lower and distributed concentration along with increased time. The numerical simulation results revealed that the ventilation arrangement would have the optimized results by utilizing case 4, on the airflow distribution, temperature distribution, pressurization, and especially on CO_2_ concentration. In addition, the baseline case is still feasible in diluting the CO_2_ concentration.

### 3.4. Concentration Contaminant Decay

**Figure 7:**
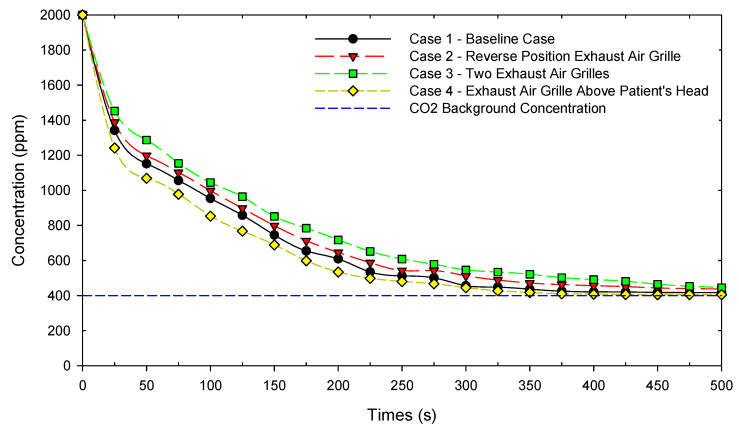
Concentration contaminant decay in different cases.

The average CO_2_ concentration was evaluated on plane XZ at 1.2 m height, which is the likely height of exhaled air from the patient. Over a 500 s simulation time has been conducted, as shown in Figure 7. The CO_2_ concentration can be reduced to 417 ppm for case 1 and 437 ppm for case 2. Even though case 2 is similar to case 1, only reversed in a position of exhaust grille, the results show that the baseline case is still better in diluting CO_2_ concentration. As for case 3, the results show that the CO_2_ concentration can be reduced to 445 ppm. In case 2 and case 3, some return air cannot be exhausted and becomes entrained back into the supply air. This is the reason why both of the cases have a higher concentration than the baseline case. The optimized result is in case 4, which can reduce CO_2_ concentration to 405 ppm. The exhaled air from the patient will directly exit, and there is no entrained return air back into the supply airstream.

### 3.5. Bioaerosol Flow Path Model

**Figure 8:**
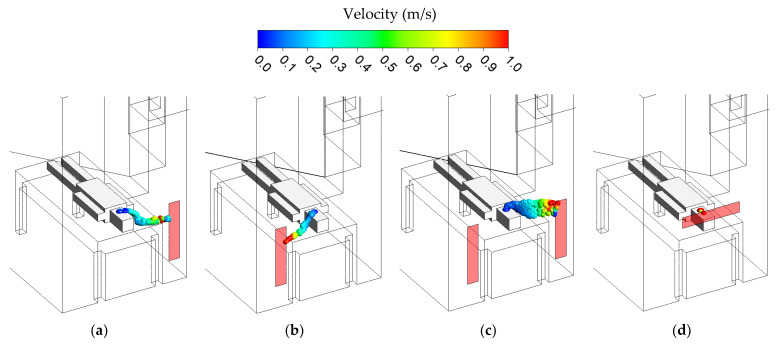
Bioaerosol flow path in different cases. (**a**) Case 1. (**b**) Case 2. (**c**) Case 3. (**d**) Case 4.

The bioaerosol model was simulated in this study with the bioaerosol model. It was injected into the spaces by the patient with the exhaled velocity of 1.12 m/s. Particle diameter sizes were 1–5 µm, and median diameter was 2.5 µm, with a density of 1000 kg/m^3^ that was approximately equal to water. The previous study also was investigated on the bioaerosol flow path in the operating room [29], which adopted the same methods and boundary conditions. In this study, the bioaerosol model was investigated in different ventilation strategies to ascertain the best approach design when facing contagious and infected patients. The bioaerosol flow path model results in different cases are illustrated in Figure 8.

Case 1 as a baseline case with an exhaust grille located 300 mm above the floor already presented a satisfactory result in terms of removing and diluting the concentration from the patient exhalation. In addition, case 2 was proposed to analyze the effect reversed side position of the exhaust air grille. The results revealed that it did not have a significant impact compared to case 1. A slightly longer particle travel time to the right corner of the isolation room in the case 2 resulted in a slightly higher concentration than case 1. Two exhaust air grilles with the same exhaust airflow rate as the baseline case were set up in case 3 in order to extract more contaminated air. However, the flow path of the bioaerosol contaminant went to the left wall of the isolation room position. It was caused by the supply air position not being in the center of the isolation room but mostly in the left area. Setting up two exhaust air grilles was avoided in this study. It would have presented a different result regarding the patient’s position when facing the right side or the left side. The airflow generated a vortex form and was more turbulent, resulting in a higher concentration. Finally, case 4, in which the exhaust air grilles above the patient’s head, presents the best position of the ventilation strategy. The exhalation from the patient was directly diluted through an exhaust air grille above the patient’s head. The flow path pattern was also shorter than the others cases. This exhaust air grille position is recommended for future isolation room design.

### 3.6. Pressurization

**Figure 9:**
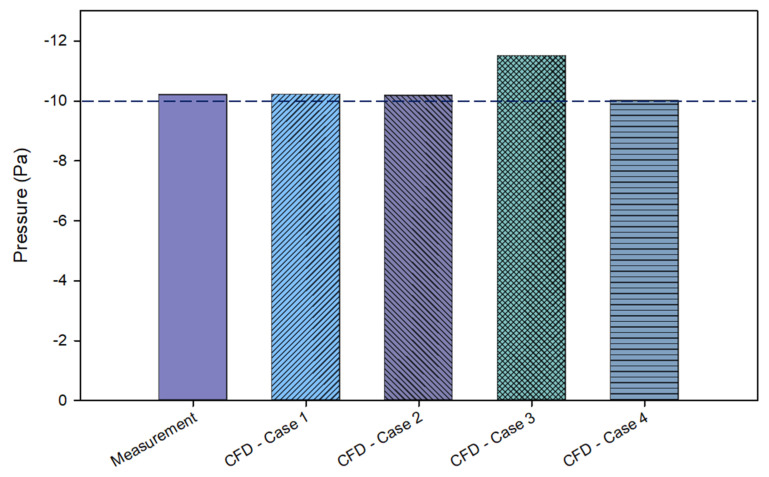
The pressurization effect in different cases.

The results of pressurization between the field measurement and numerical simulation are illustrated in Figure 9. Based on the field measurement test, it was measured that the isolation room has a pressurization of −10.2 Pa. Compared to the numerical simulation results, case 1 as a baseline case had the pressure of −10.21 Pa, which was approximately matched by the field measurement test results. The reversed side position of the exhaust air grille in case 2 also presented an insignificant difference and did not have much effect on the pressure distribution from case 1, which had pressure of −10.18 Pa. In addition, case 3 had pressure of −11.5 Pa, which revealed slightly higher negative pressure than the other cases. In this case, two exhaust air grilles placed beside the patient’s bed meant they could exhaust more air, making the negative pressure lower. Lastly, case 4 had pressure of −10 Pa. The results were slightly lower than the baseline case but still met the isolation room design requirement. Furthermore, all cases complied with the standard pressurization for the isolation room, a minimum of −10 Pa.

## 4. Conclusions

This research evaluated the performance improvement strategy of the isolation room for the emergency department. The field measurement test was carried out, and CFD simulation was numerically analyzed to improve the ventilation system. The exhaust air grille is the most important concern that directly affects the concentration distribution inside the isolation room. Finding the best layout for the ventilation system and arrangement, and diluting airborne contaminants, is also crucial. From the study, the results revealed that ventilation performance could be improved by the rearrangement of the exhaust air grille’s location. The exhaust air located above the patient’s head in case 4 revealed better ventilation performance. Its lowest concentration was around 417 ppm. It was found to be most effective at diluting the contamination approach to the background concentration levels at 400 ppm. Instead of the concentration results, it also could meet the design requirements of temperature at 22 °C, and the pressure requirements at −10 Pa within the isolation room. These findings could be provided to lead the development strategy for the effective removal of airborne contaminants in isolation rooms.

## Figures and Tables

**Table 1:** Field measurement data.

Location	Temperature (°C)	Air Flow Rate (CMH)	Air Changes Hour (ACH)	Pressure (Pa)
SA-1	18.1	291	16.76	Anteroom → E.D. −10.2
EA-1	24.0	350		

**Table 2:** Boundary condition.

Parameter	Type	Value
Supply air	Velocity inlet Discrete phase: escape	Velocity: 1.28 m/s Temperature: 18.1 °C Concentration: 400 ppm
Exhaust air	Pressure outlet Discrete phase: trap	Temperature: 24 °C Pressure: −10.0 Pa
Exhaled air by patient	Velocity inlet	Velocity inlet: 1.12 m/s Temperature: 37 °C Patient’s exhale: 45,000 ppm
Bioaerosol	DPM: Injection	Velocity: 1.5 m/s Flowrate: 0.17 kg/s Particle Size: 1–5 µm, median 2.5 µm
Patient	Wall	Heat flux: 34.87 W/m^2^

**Table 3:** Relative error percentage using different grid numbers.

Previous Grid Value	New Grid Value	Relative Error (%)
2,849,666	1,219,351	1.34
1,455,589	514,414	1.83
985,386	263,380	2.74
489,608	78,038	5.27
362,748	32,922	10.02

**Table 4:** The temperature results on each point in different cases.

Location	Case 1	Case 2	Case 3	Case 4
Point 1	19.13	19.15	19.67	19.10
Point 2	19.55	20.28	19.82	19.35
Point 3	20.25	20.52	20.65	20.24
Point 4	20.35	20.33	20.42	20.87

**Table 5:** The concentration contaminant on each point in different cases.

Location	Case 1	Case 2	Case 3	Case 4
Point 1	400.49	401.14	407.33	400.15
Point 2	468.45	471.35	478.59	432.35
Point 3	494.34	565.12	604.32	480.42
Point 4	589.25	520.3	576.33	550.15

## Data Availability

The data presented in this study are available on request from the corresponding author.
